# Decidualization and Syndecan-1 Knock Down Sensitize Endometrial Stromal Cells to Apoptosis Induced by Embryonic Stimuli

**DOI:** 10.1371/journal.pone.0121103

**Published:** 2015-04-01

**Authors:** Sarah Jean Boeddeker, Dunja Maria Baston-Buest, Tanja Fehm, Jan Kruessel, Alexandra Hess

**Affiliations:** 1 Department of Obstetrics/Gynecology and Reproductive Endocrinology and Infertility (UniKiD), Medical Center University of Duesseldorf, Duesseldorf, Germany; 2 Department of Obstetrics and Gynecology, Medical Center University of Duesseldorf, Duesseldorf, Germany; South China Agricultural University, CHINA

## Abstract

Human embryo invasion and implantation into the inner wall of the maternal uterus, the endometrium, is the pivotal process for a successful pregnancy. Whereas disruption of the endometrial epithelial layer was already correlated with the programmed cell death, the role of apoptosis of the subjacent endometrial stromal cells during implantation is indistinct. The aim was to clarify whether apoptosis plays a role in the stromal invasion and to characterize if the apoptotic susceptibility of endometrial stromal cells to embryonic stimuli is influenced by decidualization and Syndecan-1. Therefore, the immortalized human endometrial stromal cell line St-T1 was used to first generate a new cell line with a stable Syndecan-1 knock down (KdS1), and second to further decidualize the cells with progesterone. As a replacement for the ethically inapplicable embryo all cells were treated with the embryonic factors and secretion products interleukin-1β, interferon-γ, tumor necrosis factor-α, transforming growth factor-β1 and anti-Fas antibody to mimic the embryo contact. Detection of apoptosis was verified via Caspase ELISAs, PARP cleavage and Annexin V staining. Apoptosis-related proteins were investigated via antibody arrays and underlying signaling pathways were analyzed by Western blot. Non-decidualized endometrial stromal cells showed a resistance towards apoptosis which was rescinded by decidualization and Syndecan-1 knock down independent of decidualization. This was correlated with an altered expression of several pro- and anti-apoptotic proteins and connected to a higher activation of pro-survival Akt in non-differentiated St-T1 as an upstream mediator of apoptotis-related proteins. This study provides insight into the largely elusive process of implantation, proposing an important role for stromal cell apoptosis to successfully establish a pregnancy. The impact of Syndecan-1 in attenuating the apoptotic signal is particularly interesting in the light of an already described influence on pregnancy disorders and therefore might provide a useful clinical tool in the future to prevent pregnancy complications provoked by inadequate implantation.

## Introduction

In human, four days after the oocyte was fertilized in the fallopian tube, it reaches the uterus and implants into the inner wall, the endometrium, for further growth and development. Embryo invasion through first endometrial epithelial cells (EECs) and subsequent implantation into the endometrial stromaare crucial steps for a successful pregnancy. This process requires a receptive endometrium, a good quality embryo and a synchronized molecular dialogue between embryo and maternal endometrium [1]. A receptive endometrium is characterized by decidualization of endometrial stromal cells (ESCs) in response to progesterone with morphological changes of the elongated fibroblast-like cells to enlarged, rounded cells [2]. The embryo-maternal dialogue is conducted via secreted cytokines as well as expression of corresponding receptors and co-receptors [3,4]. An alleged important co-receptor for cytokines, which is highly upregulated in the receptive human endometrium is the heparan sulfate proteoglycan Syndecan-1 (Sdc-1) [5]. It is typically present on the cell surface [6], but can also accumulate in the nucleus [7] and exists in the extracellular milieu and body fluids due to proteolytical cleavage from the cell surface as well [8,9]. Hence, the supposed biological functions of Sdc-1 are rather complex and comprise regulation of cell-cell-interaction, cell migration as well as tumorigenesis and consequently attracted interest in the field of obstetrics and gynaecology as well as reproductive medicine in the recent years. Correspondingly, an altered placental Sdc-1 expression was already associated with several pregnancy complications and disorders, which in turn arise from an inadequate implantation [10–12].

The exact cellular mechanisms mediating a proper implantation in human are still not fully understood. Disruption of the endometrial epithelium was intensely investigated and particularly correlated with Fas-mediated apoptosis after binding of the Fas-ligand (FasL)-bearing embryo to the Fas-receptor (FasR) expressing endometrial cell so far [13].

Apoptosis is characterized by fragmentation and engulfing of cell compartments into membrane-covered apoptotic bodies which can be subsequently removed without any immune response or damage of the surrounding cells [14]. It is orchestrated by a cascade of caspases which can be classified in initiator caspases, like Caspase-8 and -9 at the beginning of the pathway and following effector caspases, like Caspase-3 inducing the cellular morphological changes [15]. The Inhibitor of Apoptosis (IAP) family includes different members like XIAP, cIAP-1, -2 and Livin which can bind directly to caspases and thereby inactivate them [16]. Pro-apoptotic molecules like Second mitochondria-derived activator of caspases (SMAC) and High temperature requirement protein A2 (HtrA2) bind IAPs and diminish or even prevent their inhibitory effects on apoptosis on the other side [17]. Cell interactions of pro- and anti-apoptotic molecules are well balanced and altered on demand with a shift to pro-apoptotic proteins leading to the programmed cell death.

Up to date there is little known about a possible role for ESC apoptosis as a consequence to an embryo contact. Evidence from animal studies point out that apoptosis of ESCs plays a major role in the establishment of a pregnancy [18–20] and in contrast to the fast disruption of EECs is regulated in a moderate spatial and temporal fashion [21].

Studies in human are controversial though: mostly a clear role for ESC apoptosis during menstruation is described [22] whereas its role in implantation is not comprehensive. On one hand the presence of scattered apoptotic dESCs in normal human first trimester pregnancy vs. elevated apoptotic dESCs in cases of miscarriage [23] was shown. Furthermore, a study regarding endometriosis, a disease characterized by the presence of endometrial tissue located outside the uterus, described the presence of apopotic ESCs in normal, healthy control individuals throughout the menstrual cycle, which were found to be decreased in patients suffering from endometriosis [24,25]. This is particularly intriguing since on the opposite another publication depicted a resistance of human ESCs towards apoptosis *in vitro* [26–28]. Therefore, the exact role of ESC apoptosis is ambiguous and still needs to be investigated.

An influence of Sdc-1 on apoptosis was shown before for various cancer cells and revealed a Janus-faced attitude, since in some cancer types, e.g. cervical and endometrial, a low Sdc-1 expression was correlated with high viability and low apoptosis [29,30] whereas in other cancer types, e.g. breast and ovarian, the contrary was observed [31,32].

Therefore, the aim of this study was to investigate the inducibility and regulation of apoptosis in human ESCs induced via embryonic secretion products and surface molecules as a model for embryo contact during implantation to identify the role of apoptosis in conveying a successful pregnancy. In particular, the role of decidualization affecting ESCs regarding their susceptibility to apoptosis should be evaluated. Furthermore, a conceivable influence of Sdc-1 on apoptosis was examined using a stable Sdc-1 knock down (kd) cell line to further clarify its physiological role in human implantation with regard to the regulation of apoptosis.

## Materials and Methods

### Cell lines, culture conditions and decidualization

The immortalized human endometrial stromal cell line St-T1 was a generous gift from Professor Brosens (University of Warwick, UK), whose group also generated the cell line [33] and initially characterized them for functionality and comparability to primary endometrial stromal cells [34]. St-T1 with a stable and inducible Sdc-1 kd (KdS1) were generated in our laboratory and described before [35]. Cells were maintained at 5% CO_2_ and 37°C in a mixture of 75% (v/v) DMEM and 25% (v/v) MCBD 105 supplemented with 10% (v/v) charcoal-stripped fetal calf serum (FCS), 1x penicillin/streptomycin, 40μg/ml gentamycin, 1x sodium pyruvate, 2mM L-glutamine, 1mM non-essential amino acids (all Biowest, Nuaillé, France) and 5μg/ml insulin (Sigma-Aldrich, Steelze, Germany). Cells were decidualized with 0.5mM 8-bromo-cAMP (Biolog, Bremen, Germany) and 1μM medroxyprogesterone 17-acetate (MPA; Sigma-Aldrich) for 72h. Decidualization was proven morphologically via bright-field microscope analysis and biochemically via determination of prolactin (S1 Fig.). Sdc-1 kd of KdS1 was induced applying 1μg/ml tetracycline (tet) for 48h as described before [35].

For the experiments cells were treated with 10ng/ml interleukin (IL)-1β, 10ng/ml interferon (IFN)-γ, 5ng/ml tumor necrosis factor (TNF)-α, 0.5ng/ml transforming growth factor (TGF)-β1 (all Biolegend, San Diego, CA, USA) for 24h alone and in combination (IITT), with 5μg/ml anti-human Fas antibody (ab) clone EOS9.1 (F) (Biolegend) for 24h alone and after 24h pretreatment with IITT (IITT+F) as a replacement for the embryos secretome as established in our laboratory before [36].

### Active Caspase-3 measurement

Expression of active Caspase-3 was analyzed applying the Quantikine ELISA (R&D Systems). Non-differentiated St-T1 and KdS1 as well as decidualized St-T1 (dSt-T1) and KdS1 (dKdS1) grown in 24-well plates were treated as described above (n = 3 each). Cell extract preparation and measurement of active Caspase-3 was performed according to the manufacturer’s protocol and given as relative amount of active Caspase-3 in ng/ml.

### Western blot analysis of poly (ADP-ribose) polymerase (PARP)

Non-differentiated St-T1 and KdS1 as well as dSt-T1 and dKdS1 (n = 3 each) were treated with IITT+F as described above and protein lysates were prepared using Cell Lysis Buffer (Cell Signaling Technology, Danvers, MA, USA) according to the manufacturer’s protocol. Briefly, 30μg protein was separated by 12% SDS-PAGE and transferred to a PVDF membrane (Merck Millipore). The membrane was blocked with 5% non-fat milk and incubated with antibodies against PARP 1:1000 (9542, Cell Signaling Technology) and β-Actin 1:5000 (ab6276, Abcam, Cambridge, UK) at 4°C over night, followed by goat anti-rabbit HRP ab 1:2000 (R&D Systems) for 1h. Signals were detected using Clarity Western ECL Substrate (Bio-Rad, Hercules, CA; USA) and analyzed with the ChemiDoc Imaging System (Bio-Rad) and the corresponding Image Lab software.

### Immunfluorescence of Annexin V

Phosphatidylserine-translocation in apoptotic cells was visualized via FITC Annexin V staining of non-differentiated St-T1 and KdS1 as well as dSt-T1 and dKdS1 grown in 35mm dishes (n = 3 each), treated with IITT+F as described above and compared to untreated cells. Cells were washed with PBS containing 2% (v/v) FCS and 0.09% (v/v) sodium azide twice and stained with FITC Annexin V (Bioloegend) diluted 1:20 and 1μg/ml Hoechst 33342 (Sigma-Aldrich) in Annexin V Binding Buffer (Biolegend) for 15min and analyzed with a Zeiss Axiovert 200 microscope and the AxioVision software (Zeiss, Oberkochen, Germany).

### Active Caspase-8 and -9 measurements

Non-differentiated St-T1 and KdS1 as well as dSt-T1 and dKdS1 were treated with IITT+F as described above and compared to non-treated controls (n = 5 each). Enzymatic activity of the Caspases-8 and -9 was determined via the corresponding Colorimetric Assay Kit (R&D Systems) according to the manufacturer’s manual.

### Profiling of apoptosis-related proteins

The expression of 35 different apoptosis-related proteins of total protein lysate was analyzed via the Proteome Profiler Human Apoptosis Array Kit (R&D Systems). Briefly, non-differentiated St-T1 and KdS1 as well as dSt-T1 and dKdS1 (n = 4 each) were first analyzed without a treatment to identify the baseline expression of apoptosis-related proteins and second after treatment with IITT+F as described above. Cell extract preparation for the array with 400μg protein was performed according to the manufacturer’s protocol. Dot blots were photographed and analyzed with an Alpha Imager Camera and the corresponding AlphaView Software (Biozym Scientific GmbH, Hessisch Oldendorf, Germany)

### FasR analysis

Non-differentiated St-T1 and KdS1 as well as dSt-T1 and dKdS1 were treated with IITT 24h and compared to non-treated controls (n = 5 each). Induction of FasR after treatment with IITT was investigated by real time PCR using the 2^-ΔΔCt method [37]. Primers for FasR were for 5’-GGACCCTCCTACCTCTGGTTCTTA-3’ and rev 5’-TTCACCTGGAGGACAGGGCTT-3’ (annealing temperature 50°C) and were normalized to the housekeeping gene β-Actin; primers were 5’-CGGGACCTGACTGACTACC-3’ and rev 5’-AGGAAGGCTGGAAGAGTGC-3’ (annealing temperature 50°C).

### Western blot analysis of Akt, JNK, p65 and IκBα

Non-differentiated St-T1 and KdS1 as well as dSt-T1 and dKdS1 were treated with IITT for 15min, F for 15min as well as IITT 24h + F for 15min and compared to untreated controls (n = 6 each). Experiments to determine a time dependent expression were performed in our laboratory before. Protein lysates were prepared with Cell Lysis Buffer (Cell Signaling Technology) according to the manufacturer`s protocol. 30μg protein was separated by 12% SDS-PAGE and transferred to a PVDF membrane (Merck Millipore). The membrane was blocked with 5% non-fat milk and incubated with antibodies against Akt (9542), phosphorylated (p)Akt (4060), JNK (9258), pJNK (4668), p65 (8242), pp65 (3039) IκBα (4814) all 1:1000 (all Cell Signaling Technology) and β-Actin (ab6276, Abcam) at 4°C over night, followed by goat anti-rabbit or goat anti-mouse HRP ab 1:2000 (R&D Systems) for 1h. Signals were detected using Clarity Western ECL Substrate (Bio-Rad) and analyzed with the ChemiDoc Imaging System (Bio-Rad) and the corresponding Image Lab software.

### Statistical analysis

Two groups each were analyzed applying unpaired 2-tailed Mann-Whitney-U-Tests, and ANOVAs with Bonferroni post hoc tests were used for multiple comparisons by using SPSS 22 (SPSS, Chicago, IL, USA). Values of p<0.05 were considered statistically significant.

## Results

### Quantification of active Caspase-3 in ESCs treated with embryonic stimuli

**Fig 1:**
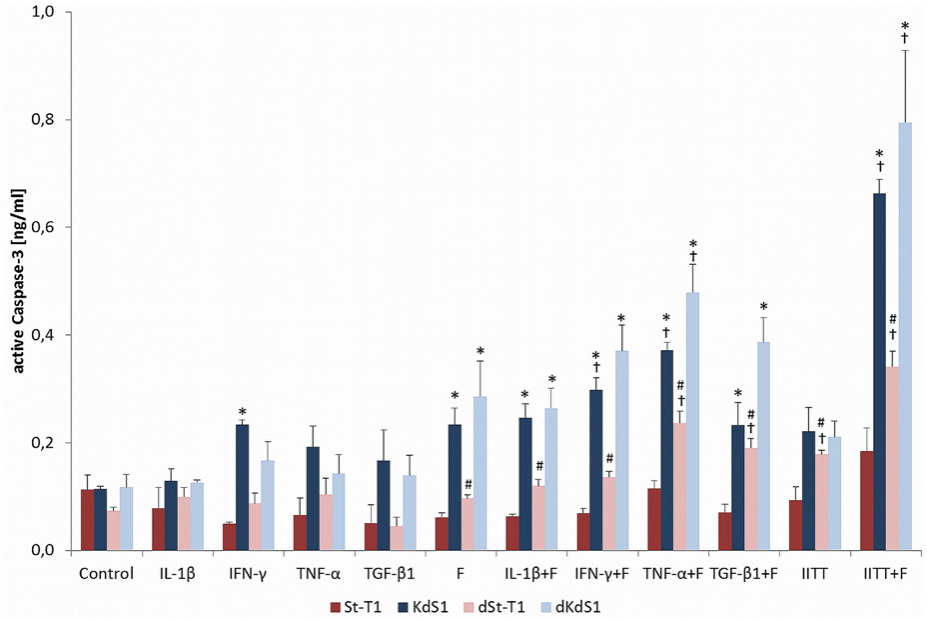
Quantification of active Caspase-3 in ESCs treated with embryonic stimuli. Non-differentiated (St-T1, red bar; KdS1, blue bar) and decidualized (dSt-T1, bright red bar; dKdS1, bright blue bar) ESCs were treated with IITT and F for 24h individually or in combination as indicated and the amount of active Caspase-3 was analyzed in ng/ml and displayed as mean±SEM of n = 3 independent experiments; *p<0.05 Sdc-1 wildtype vs. Sdc-1 kd cells, #p<0.05 non-differentiated vs. decidualized cells, ✝p<0.05 untreated controls vs. treated cells.

First, induction of apoptosis was quantified by investigating the total amount of active Caspase-3 after treatment with IITT and F separately or in combination (Fig. 1). Non-differentiated St-T1 showed no significant increase of active Caspase-3 compared to untreated control and consequently no induction of apoptosis after all tested treatments. In contrast KdS1 revealed a significant increase of active Caspase-3 compared to control after treatment with combination of IFN-γ+F as well as TGF-β1+F and combination of IITT+F. In addition dSt-T1 induced apoptosis after treatment with TNF-α+F, TGF-β1+F, IITT and IITT+F whereas dKdS1 displayed apoptosis induction after treatment with TNF-α+F and likewise IITT+F. The latter incubation condition led to the highest increase of active Caspase-3 in all cells except non-differentiated St-T1 with a significant raise due to the Sdc-1 kd (St-T1 vs. KdS1 and dSt-T1 vs. dKdS1) and according to decidualization (St-T1 vs. dS-T1 and KdS1 vs. dKdS1).

### PARP-cleavage upon treatment with embryonic stimuli

**Fig 2:**
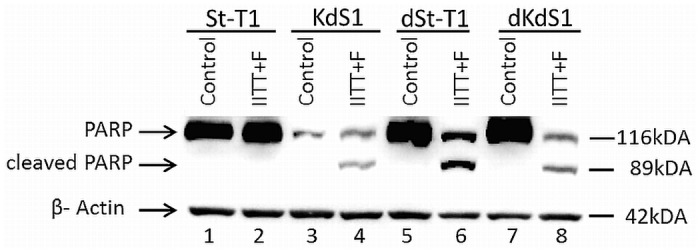
PARP-cleavage upon treatment with embryonic stimuli. Representative Western Blot analysis of PARP cleavage (full length PARP 116kDa; cleaved PARP 89 kDa) in non-differentiated (St-T1, lanes 1+2; KdS1, lanes 3+4) and decidualized (dSt-T1, lanes 5+6; dKdS1, lanes 7+8) ESCs after treatment with IITT+F vs. untreated controls, n = 3; β-Actin (42kDa) served as a loading control.

After treatment of cells with IITT+F cleavage of PARP vs. untreated controls as the main downstream target of active Caspase-3 was shown via Western Blot analysis in KdS1, dSt-T1 and dKdS1 (Fig. 2), whereas no cleavage was seen in St-T1.

### Loss of membrane asymmetry after IITT+F treatment

**Fig 3:**
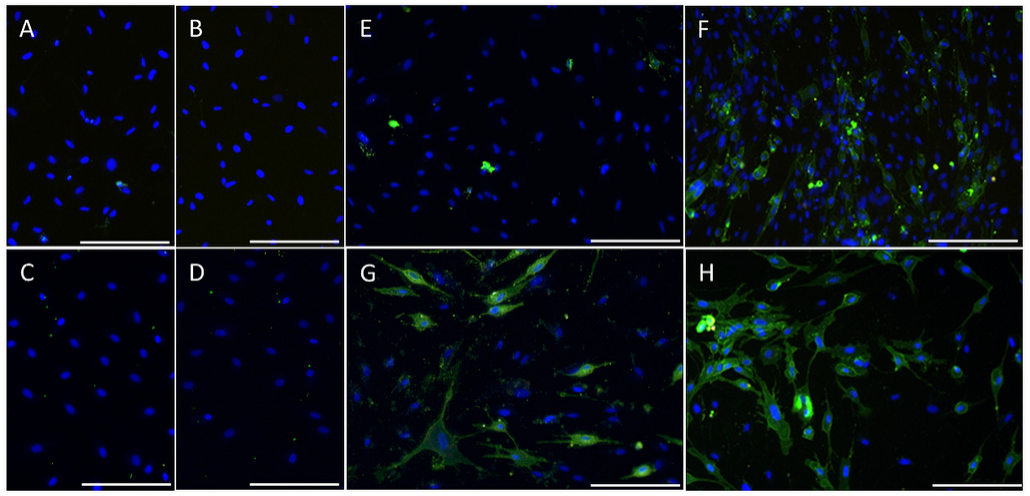
Loss of membrane asymmetry after IITT+F treatment. Non-differentiated (St-T1, KdS1) and decidualized (dSt-T1, dKdS1) ESCs were treatmed with IITT+F and loss of membrane asymmetry was visualized with Annexin V FITC staining (green), n = 3; (A)–(D) untreated controls: (A) St-T1, (B) KdS1, (C) dSt-T1, (D) dKdS1. (E)-(H) IITT+F treated: (E) St-T1, (F) KdS1, (G) dSt-T1, (H) dKdS1; blue nuclei are stained with Hoechst 33342. Scale bars indicate 100μm.

The loss of membrane asymmetry with a phosphatidylserine-translocation from the inner to the outer cell membrane as a consequence of apoptosis was investigated by staining of cells with Annexin V after treatment with IITT+F. KdS1 (Fig. 3F), dSt-T1 (Fig. 3G) and dKdS1 (Fig. 3H) showed a green staining whereas none was detectable in the controls (Fig. 3A–3D) and IITT+F treated St-T1 (Fig. 3E). In the latter only a light green staining was observed possibly indicating spontaneous apoptosis. The bright green spots without a cell shape display artefacts.

### Investigation of the extrinsic and intrinsic apoptosis pathway

**Fig 4:**
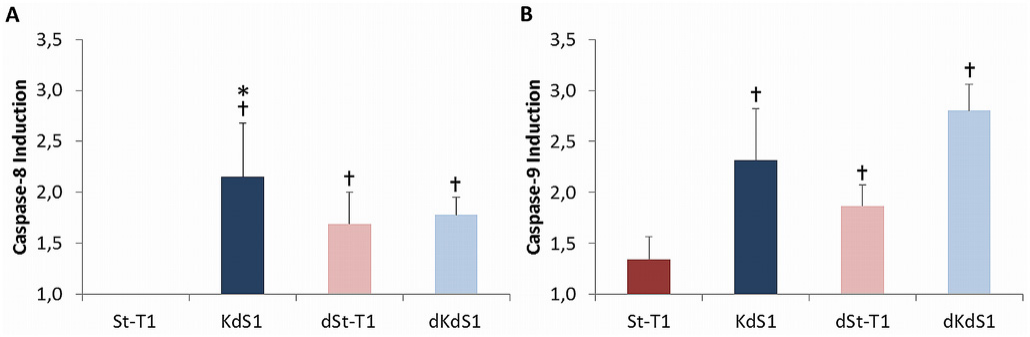
Investigation of the extrinsic and intrinsic apoptosis pathway after IITT+F treatment. Analysis of Caspase-8 and -9 activation of treated cells vs. untreated controls in non-differentiated (St-T1, red bar; KdS1, blue bar) and decidualized dSt-T1, bright red bar; dKdS1, bright blue bar) ESCs after treatment with IITT+F. Untreated controls were assigned being 1 and enzymatic activity of caspases after treatment was determined as fold induction vs. controls and given as mean±SEM of n = 3 independent experiments, *p<0.05 Sdc-1 wildtype vs. Sdc-1 kd cells, ✝p<0.05 untreated controls vs. treated.

To study the activation of pro-Caspase-8 and -9, incubation with IITT+F was chosen. Non-differentiated KdS1, dSt-T1 and dKdS1 showed a significant induction of Caspase-8 (Fig. 4A) and -9 (Fig. 4B) after treatment when compared to untreated controls which was assigned 1. No Caspase-8 induction was found for treated St-T1 vs. untreated control and for Caspase-9 only a slight but not statistically significant induction was observed. Within the group of treated cells (St-T1, KdS1, dSt-T1, dKdS1), only Caspase-8 induction was significantly higher in KdS1 compared to St-T1.

### Expression of apoptosis-related proteins before and after IITT+F treatment

**Fig 5:**
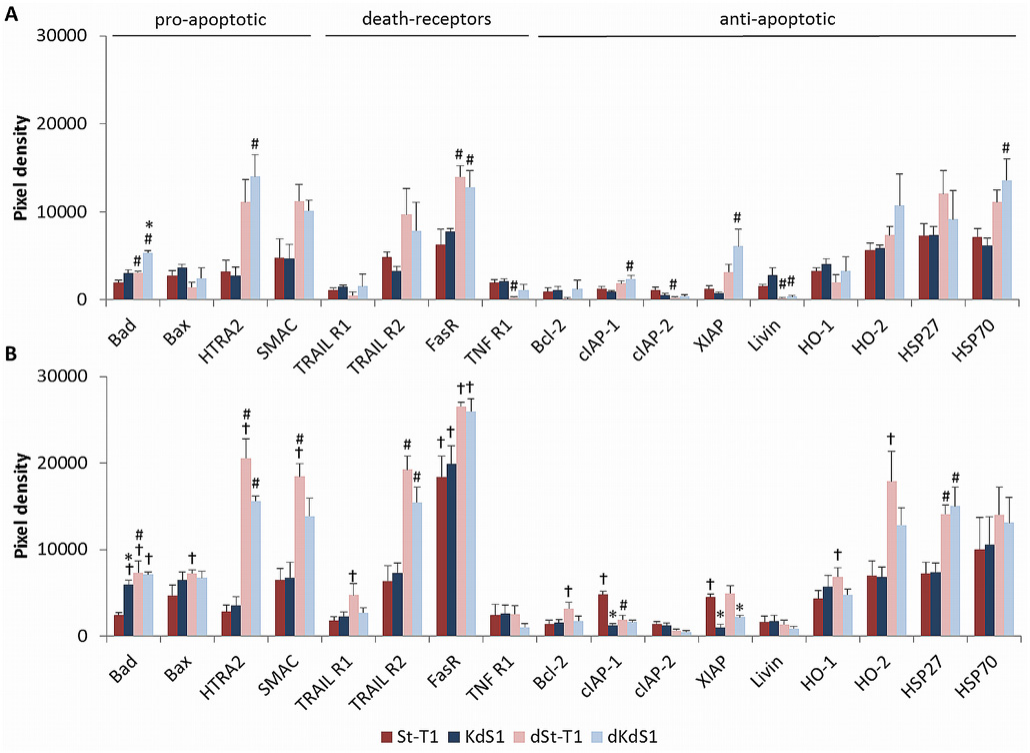
Expression of apoptosis-related proteins before and after IITT+F treatment. Antibody array analysis of apoptosis-related proteins in ESCs of (A) untreated, non-differentiated (St-T1, red bar; KdS1, blue bar) and decidualized (dSt-T1, bright red bar; dKdS1, bright blue bar) ESCs, n = 4 ± SEM, *p<0.05 Sdc-1 wildtype vs. Sdc-1 kd cells, #p<0.05 non-differentiated vs. decidualized cells. (B) Antibody Array with protein from IITT+F treated, non-differentiated (St-T1, red bar; KdS1, blue bar) and decidualized (dSt-T1, bright red bar; dKdS1, bright blue bar) ESCs. Pixel density is given as mean±SEM of n = 4 independent experiments, *p<0.05 Sdc-1 wildtype vs. Sdc-1 kd cells, #p<0.05 non-differentiated vs. decidualized cells, ✝p<0.05 untreated vs. IITT+F treated.

The analysis of apoptosis-related proteins in untreated cells as a baseline expression (Fig. 5A) demonstrated a statistically significant increase of pro-apoptotic Bad in accordance to decidualization and additive to the Sdc-1 kd. The death receptor FasR was also increased in both decidualized cell types dSt-T1 and dKdS1, whereas anti-apoptotic Livin was decreased. Pro-apoptotic HTRA2 and anti-apoptotic cIAP-1, XIAP and heat shock protein (HSP) 70 were increased in dKdS1 compared to non-differentiated KdS1, while cIAP-2 and TNF R1 were decreased in dSt-T1 compared to St-T1.

Fig. 5B shows that treatment with IITT+F led to a significant upregulation of several proteins: pro-apoptotic Bad in KdS1, dSt-T1 and dKdS1; Bax, HTRA2, SMAC, TNF-related apoptosis-inducing ligand receptor (TRAIL R1) and anti-apoptotic Bcl-2, heme oxygenase (HO)-1 and -2 in dSt-T1. FasR was significantly upregulated in all cell types, whereas anti-apoptotic cIAP-1 and XIAP increased in St-T1. As a consequence Bad was significantly higher in accordance to the Sdc-1 kd in KdS1 whereas cIAP-1 and XIAP were significantly lower. Bad, HTRA2, SMAC, TRAIL R2 and HSP27 were significantly higher dependent on decidualization, while cIAP-1 was downregulated in dSt-T1 compared to St-T1.

### Induction of FasR expression after IITT

**Fig 6:**
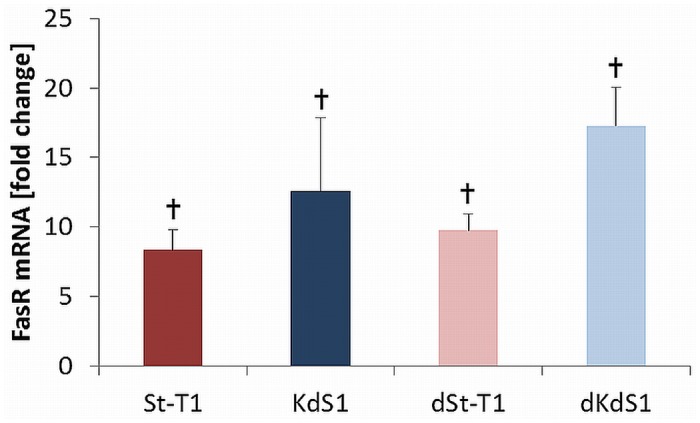
Induction of FasR expression after IITT treatment. Fold change of FasR mRNA in non-differentiated (St-T1, red bar; KdS1, blue bar) and decidualized (dSt-T1, bright red bar; dKdS1, bright blue bar) ESCs after treatment with IITT 24h. 2^-ΔΔCt^ is are given as mean±SEM of n = 5 independent experiments, ✝p<0.05 untreated vs. IITT treated.

FasR protein was significantly upregulated in all investigated cell types after IITT+F treatment (Fig. 5B). *Real time* PCR analysis showed FasR mRNA was already upregulated after single treatment with IITT for 24h without F (Fig. 6).

### Analysis of apoptosis-related signaling pathways

**Fig 7:**
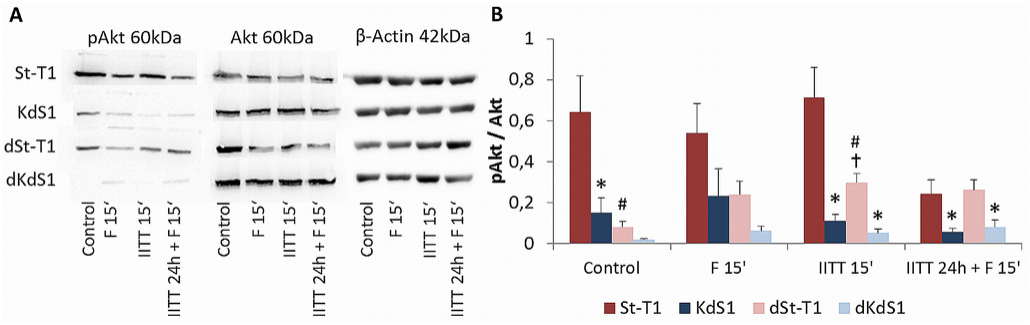
Activation of the pro-survival protein Akt after IITT and F treatment. Western blot analysis of pAkt and Akt in non-differentiated (St-T1, first line; KdS1, second line) and decidualized (dSt-T1, third line; dKdS1, fourth line) ESCs after treatments with F 15min, IITT 15min and IITT 24h + F 15min vs. untreated controls. (A) Representative blot of pAkt (60kDa), Akt (60kDa) and β-Actin (42kDa) as loading control. (B) Pixel densitiy evaluation of pAkt normalized to Akt is given as mean±SEM of n = 6 independent experiments, *p<0.05 wildtype vs. Sdc-1 kd cells, #p<0.05 undifferentiated vs. decidualized cells, ✝p<0.05 untreated control vs. treated.

Short time incubation treatments for 15min were performed to analyze a possible activation of the cell signaling proteins Akt, nuclear factor (NF)κB members p65 and inhibitor of kappa B (IκBα) as well as mitogen-activated protein kinase (MAPK) and c-Jun N-terminal kinase (JNK). Fig. 7 illustrates that Akt was constitutively active in non-differentiated St-T1. Interestingly, the Sdc-1 kd in KdS1 or decidualization in dSt-T1 led to a decrease of pAkt. Furthermore a strong additive decrease of pAkt in dKdS1 was observed. Correspondingly, the pixel density analysis revealed a significant decrease compared to St-T1 in the controls and after short treatment with IITT 15min (Fig. 7B).

**Fig 8:**
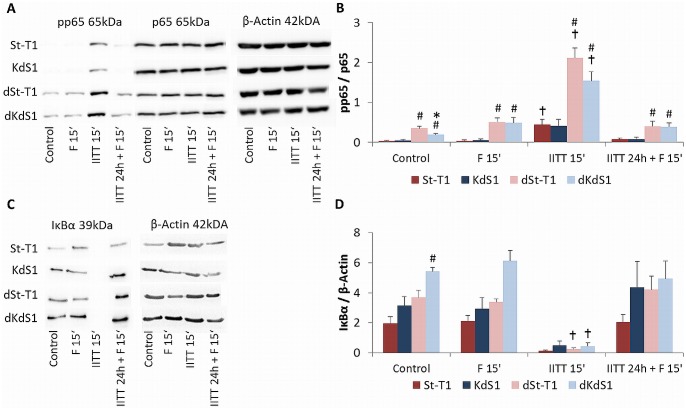
Activation of the pro-survival NFκB pathway after IITT and F treatment. Western blot analysis of NFκB members p65 and IκBα in non-differentiated (St-T1, first line; KdS1, second line) and decidualized (dSt-T1, third line; dKdS1, fourth line) ESCs after treatments with F 15min, IITT 15min and IITT 24h + F 15min vs. untreated controls. (A) Representative blot of pp65 (65kDa), p65 (65kDa) and β-Actin (42kDa) as loading control. (B) Pixel densitiy evaluation of pp65 normalized to p65 is given as mean±SEM of n = 6 independent experiments, *p<0.05 wildtype vs. Sdc-1 kd cells, #p<0.05 undifferentiated vs. decidualized cells, ✝p<0.05 untreated control vs. treated. (C) Representative blot of IκBα (39kDa) and β-Actin (42kDa) as loading control. (D) Pixel densitiy evaluation of IκBα normalized to β-Actin is given as mean±SEM of n = 6 independent experiments, *p<0.05 wildtype vs. Sdc-1 kd cells, #p<0.05 undifferentiated vs. decidualized cells, ✝p<0.05 untreated control vs. treated.

The NFκB member pp65 (Fig. 8A and 8B) as an indirect downstream target of pAkt was not upregulated in non-differentiated St-T1, whereas pp65 rather significantly increased in response to decidualization in dSt-T1 and dKdS1 vs. non-differentiated cells. Furthermore, a strong induction after IITT treatment for 15min led to a significant increase of pp65 compared to control. Accordingly, the corresponding inhibitor IκBα (Fig. 8C and 8D) was downregulated after IITT treatment for 15min, but no differences between non-differentiated and decidualized stromal cells were observed.

**Fig 9:**
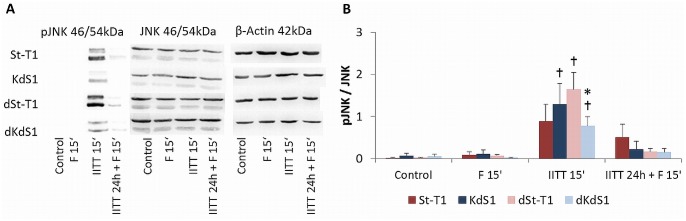
Activation of the pro-apoptotic JNK pathway after IITT and F treatment. Western blot analysis of pJNK and JNK in non-differentiated (St-T1, first line; KdS1, second line) and decidualized (dSt-T1, third line; dKdS1, fourth line) ESCs after treatments with F 15min, IITT 15min and IITT 24h + F 15min vs. untreated controls. (A) Representative blot of pJNK (46/54kDa), JNK (46/54kDa) and β-Actin (42kDa) as loading control. (b) Pixel densitiy evaluation of pJNK normalized to JNK is given as mean±SEM of n = 6 independent experiments, *p<0.05 wildtype vs. Sdc-1 kd cells, ✝p<0.05 untreated control vs. treated.

Finally Fig. 9A and 9B depict that pJNK as a common mediator of apoptosis is significantly increased in KdS1, dSt-T1 and dKdS1 after treatment with IITT for 15min, whereas no statistically significant increase was observed for St-T1 treated with IITT for 15min.

## Discussion

The attachment of an embryo to the maternal endometrial epithelium and its consequent invasion in first the endometrial epithelium and second the endometrial stroma is an indispensable prerequisite for a successful pregnancy. Although it is described that cell death of EECs is the *modus operandi* how the embryos way into the stroma is facilitated [13], little is known about the role of apoptosis within ESCs to achieve the appropriate invasion depth for the implanting embryo. If anything, there are rather controversial studies describing both presence and resistance of ESCs towards apoptosis [26,38].

Hence, in the present study the competence of embryonic stimuli (IITT+F) to mimic the situation of embryo implantation regarding their apoptotic competence in human ESCs was fathomed closer. The aim was to achieve detailed information about the possible role for ESC apoptosis during the embryo implantation process. Furthermore, the influence of Sdc-1 on the apoptotic signal and expression of apoptosis-related proteins was investigated using a stable Sdc-1 kd cell line. Since we have shown before that Sdc-1 modulates apoptosis in EECs revealing an increase of apoptotic sensitivity towards IITT+F in connection with a downregulation of anti-apoptotic proteins and upregulation of the death receptors FasR and TRAIL R 2 by Sdc-1 kd [36]. This study is limited to *in vitro* experiments with immortalized cell lines, to achieve reproducible insights of Sdc-1’s influences using a stable knock down. Furthermore, treatment of cells with 4 cytokines (IITT) and anti-Fas ab (F) was applied as a replacement to mimic embryo contact in the absence of a more suitable equivalent, as described previously [36]. In detail IL-1β was chosen because it is highly secreted by human embryos *in vitro* [39,40]. Furthermore IFN-γ is also expressed by the early embryo and uterine immune cells which rapidly expand in early pregnancy [41]. TNF-α and the corresponding receptors are expressed in the endometrium, placenta and the fetus during pregnancy [42]. TGF-β1 is expressed in endometrial stromal cells [43], in the early embryo [44] and in the placenta [45].

Studies in endometrial cancer showed that upregulated Sdc-1 promotes the viability of the cells via activation of NFκB [29] whereas silencing of Sdc-1 induced apoptosis correlated with a decrease in the activation of MAPK Erk and Akt [46]. Additionally, Sdc-1 kd was shown to enhance the sensitivity to TRAIL-induced apoptosis [47]. Hence, in spite of the contrary literature, an influence of Sdc-1 kd in ESC apoptosis by modulation of MAPK, Akt and NFκB or death receptor signaling is conceivable although up to date the knowledge about the exact mechanism is very limited.

Clinical studies correlated an altered Sdc-1 expression with the risk of pregnancy diseases like preeclampsia, fetal growth restriction and preterm delivery, pregnancy complications which in turn are allocated most likely due to insufficient invasion [10–12,48]. Therefore, we hypothesized that Sdc-1 facilitates a proper implantation via regulating maternal cell apoptosis as a reaction to a contact with the embryo and its secretome.

The ESCs used in this study were able to be decidualized *in vitro* in the presence of cAMP and MPA. Consequently, four different cell conditions (non-differentiated with and without Sdc-1 kd and decidualized with and without Sdc-1 kd) were generated for all experiments. Analyzing the inducibility of apoptosis in reaction to treatment with IITT+F, the non-differentiated St-T1 revealed a resistance towards apoptosis under all tested incubation conditions. In more detail, no significant increase of Caspase-3 activation after treatment with IITT+F was detectable in St-T1 and consequently no PARP cleavage as the main downstream target of active Caspase-3 and a marker for cells undergoing irreversible apoptosis [49]. Additionally, there was no loss of membrane asymmetry illustrated via Annexin V staining emphasizing the St-T1 resistance towards apoptosis. Decidualization though seemed to sensitize the stromal cells for IITT+F mediated apoptosis. Interestingly, the Sdc-1 kd independent of decidualization rendered the cells for apoptosis as well. Subsequently, decidualized stromal cells with Sdc-1 kd displayed an even greater magnitude of apoptosis induction, leading to the highest active Caspase-3 activation in dKdS1. In accordance with the Caspase-3 activation, cleavage of PARP and Annexin V staining were also observed in KdS1, dSt-T1 and dKdS1 after treatment with IITT+F.

The apoptotic process can be started by two major ways: extrinsic via death receptors, like FasR or TRAIL R, mediated by Caspase-8 [50] or intrinsic by permeabilization of mitochondria and activation of Caspase-9. Both pathways administrate the activation of Caspase-3. To decipher whether the extrinsic or intrinsic apoptotic pathway is initiated via IITT+F treatment, the induction of corresponding caspases was analyzed, indicating an equivalent activation of both Caspase-8 and -9 after treatment with IITT+F in KdS1, dSt-T1 and dKdS1. Therefore, we assumed that Caspase-8 is activated as a consequence of F treatment which binds and activates correspondingly FasR, whereas Caspase-9 activation is possibly a secondary effect after Caspase-8 induced cleavage of the protein BID (BH3 interacting domain death agonist) which as a consequence initiates mitochondrial damage [51].

To find out, which apoptosis-related proteins are involved in regulating the divergence in apoptotic susceptibility, antibody arrays were conducted. The basal protein expression without induction of apoptosis revealed an increase of pro-apoptotic Bad in both decidualized cell types dSt-T1 and dKdS1 compared to the non-differentiated cells and therewith suggests a higher apoptotic susceptibility in decidualized cells even without further treatment. Additionally, pro-apoptotic Bad was further upregulated in dKdS1 compared to dSt-T1, which might result from an additive effect of decidualization and Sdc-1 kd. FasR also increased in accordance to decidualization, whilst anti-apoptotic Livin decreased due to decidualization in dSt-T1 and dKdS1. Data of upregulated stromal FasR expression in secretory compared to proliferative phase endometrium proposed a preparation of the stromal cells for the arriving FasL-bearing embryo [52].

However, IITT+F treatment provoked an increase of abundant proteins in all different cell types in varying degrees. Bad was upregulated in KdS1, dSt-T1 and dKdS1 and significantly higher compared to St-T1, whereas anti-apoptotic cIAP-1 and XIAP were significantly increased in St-T1. CIAP-1 and -2 and XIAP are members of the IAP family, which serve as suppressors of Fas-mediated apoptosis through direct Caspase-3 inhibition and modulation of the transcription factor NFκB [53]. Furthermore, XIAP is known to mediate first trimester trophoblast resistance to Fas-mediated apoptosis regardless of expressing both FasR and FasL [54], which must be kept in mind since dESCs also express both FasR and FasL [52]. Interestingly, FasR was increased in all cell types treated with IITT+F and supplemental experiments revealed, that FasR was already induced by single treatment with IITT on mRNA level, which might sensitize the cells to Fas-mediated apoptosis and showed a possibly important role for embryo-maternal FasL/FasR interaction in ESCs, as we have already described it for EECs before [36]. FasR was upregulated even in apoptosis resistant St-T1, but obviously there was no transfer of the apoptotic signal from the receptor to Caspase-3 since it was unchanged, which probably resulted from the influence of upregulated anti-apoptotic proteins specified before. It was described in the literature that pre-treatment with TNF-α/IFN-γ sensitized primary cultured ESCs to Fas-mediated apoptosis, which were resistant to single Fas-mediated apoptosis before. This was accompanied by an increase of FasR as well as an induction of Caspase-3, -8 and -9. In contrast to our findings apoptosis was induced in both non-differentiated and decidualized ESCs and there was no difference in FasR expression between ESCs and dESCs before and after TNF-α and IFN-γ treatment [28]. This discrepancy might result from the usage of different cell types, concentrations of cytokines and Fas Ab, as well as different Fas Ab clones and different detection methods applied. In our experiments another death-receptor, TRAIL R2, was significantly upregulated in IITT+F treated and decidualized cells, which indicates an additional role for the TRAIL/TRAIL R system besides FasL/FasR in implantation correlated apoptosis. It is already described that even the ligand TRAIL is upregulated upon *in vitro* decidualization [55] and expressed in dESCs in first trimester placental biopsies combined with the corresponding receptors TRAIL R1 and 2 [56]. To gain a deeper insight into signaling pathways regulating the observed apoptosis, Western blot analysis was performed to identify signaling molecules with a supposable impact on regulating the apoptotic signal in ESCs. Short time incubation with IITT, F alone and in combination was chosen, because the investigated signaling pathways are regulated quickly within 15min. In St-T1 the protein Akt was shown to be constitutively phosphorylated. A significant decrease due to the Sdc-1 kd and decidualization in the untreated controls and after IITT treatment was observed. Akt is a common mediator of viability, inhibiting the expression of pro-apoptotic proteins like Bad and promoting expression of anti-apoptotic protein like IAPs. Hence, the significant increase of anti-apoptotic proteins in St-T1, as well as the increase of pro-apoptotic Bad in KdS1, dSt-T1 und dKdS1 detected in the antibody arrays might be a consequence of increased Akt-activation in St-T1 as seen in this study. An influence of Sdc-1 on Akt-signaling was already described for endometrial cancer cells as aforementioned. Furthermore, it is known that Sdc-1 promotes HGF/Met signaling, resulting in enhanced activation of signaling pathways involved in the control of cell proliferation und survival, like RAS/MAPK and PI3K/Akt [57]. It was also described, that primary cultured ESCs constitutively secreted HGF *in vitro* [58]. Therefore, increased Akt activation in St-T1 could be regulated in an autocrine manner via secreted HGF, activation of Met and consequently Akt orchestrated by Sdc-1. Consequently, Sdc-1 missing KdS1 display a decreased pAkt level leading to higher apoptotic susceptibility as observed in this study. Decrease of pAkt upon decidualization was already described indirectly for primary ESCs [59], because it was shown that PTEN, functioning as an inhibitor of the PI3K/Akt pathway increased in dESCs during the late secretory phase [60]. Forkhead box protein O1 (FoxO1a) is a transcription factor in the cell nucleus, which participates in the induction of apoptosis [61]. Moreover, phosphorylation of FoxO1a via pAkt leads to an exclusion of FoxO from the nucleus to the cytoplasm, which is associated with reduced transcriptional activity. It is described that unphosphorylated FoxO1a accumulates in the nuclei of dESCs *in vivo* and this is correlated with a suppression of PI3K/Akt pathway upon decidualization [62]. Thus, decreased pAkt in dSt-T1 compared to St-T1 might also be a consequence to decidualization in our study.

Subsequently, two members of the NFκB protein family, p65 and IκBα, as indirect targets of pAkt were analyzed. A significant increase of pp65 in all decidualized cells with a strong induction after IITT treatment was detected but no increase in undifferentiated St-T1 as a consequence of increased Akt activation. Therefore, it seems as if Akt developed its anti-apoptotic properties via modifying other pro- and anti-apoptotic proteins besides NFκB. Consistent with the increase of pp65, the p65 inhibitor IκBα was degraded after IITT treatment, even though the difference between non-differentiated and decidualized cells as found for pp65 was not statistical significant for IκBα. A possible reason could be that only one of four members of the IκB family was studied.

Finally, the MAPKs JNK1/2 were investigated and again a significant increase in KdS1, dSt-T1 and dKdS1 after IITT treatment was seen compared to the untreated controls. In contrast, pJNK was not increased in St-T1. This indicates that apoptosis in KdS1, dSt-T1 and dKdS1 is potentially mediated via pJNK.

Taken together, *in vitro* cultured ESCs treated with IITT+F showed a resistance toward apoptosis, mediated via constitutive active Akt. Decidualization of these cells abrogated the resistance in connection with a decrease of pAkt and correspondingly an altered expression of Akt-regulated pro- and anti-apoptotic proteins. Furthermore, the Sdc-1 kd sensitized St-T1 to apoptosis independent of decidualization and in all apoptosis-sensitive cells the highest induction of Caspase-3 was measured after a combined treatment of IITT+F.

Hypothetical mechanisms being subject to the influence of Sdc-1 on ESC apoptosis might be regulating signaling pathways like MAPK or PI3K via the mediator Akt and the corresponding pro- and anti-apoptotic proteins as well as the transcription factor FoxO1a indirectly by modulation of cytokine and death receptor binding to their corresponding ligands. Additionally, a direct influence on the extrinsic death-receptor mediated pathway or on the Akt-pathway itself is conceivable.

In summary, decidualization which is necessary for proper embryo implantation provides the opportunity for the endometrial stromal cell line St-T1 to induce apoptosis in reaction to embryonic stimuli *in vitro*, which provides an indication for ESC apoptosis as an important process during implantation *in vivo*. Even if the exact Sdc-1 influence on apoptosis induction is not fully understood so far the overall findings of this study indicate that Sdc-1 may act as a modulator of ESC apoptosis and probably invasion depth as a crucial factor for successful pregnancy. Hence, the important role of Sdc-1 in influencing implantation which has been discussed extensively already and the background that the pathophysiology of preeclampsia and fetal growth restriction is correlated with an altered Sdc-1 expression on the one hand [12,48,63] and increased placental cell apoptosis on the other [64,65] supports our thesis that Sdc-1 exerts its influence on implantation via regulating ESC apoptosis.

## Supporting Information

S1 Fig Proof of decidualization markers. Representative bright-field microscope analysis of cell morphology before and after decidualization *in vitro*: (A) non-differentiated St-T1, (B) dSt-T1 after decidualization via MPA and cAMP treatment for 72h; scale bar indicates 100μm. (C) representative PCR gel for the housekeeping gene HMBS (64bp) in St-T1 (lane 1+2), KdS1 (lane 3+4), dSt-T1 (lane 5+6) and dKdS1 (lane 7+8). (D) representative PCR gel for the decidualization marker PRL (247bp) in dSt-T1 (lane 5+6) and dKdS1 (lane 7+8) vs. non differentiated St-T1 (lane 1+2) and KdS1 (lane 3+4); M = marker and (-) negative control without template. (E) quantification of secreted PRL via ELISA in in non-differentiated (St-T1, red bar; KdS1, blue bar) and decidualized (dSt-T1, bright red bar; dKdS1, bright blue bar) ESCs. Values are given as mean±SEM of n = 3 replicates.
(TIF)
