# Supplementary figures and images for: Decidualization and Syndecan-1 Knock Down Sensitize Endometrial Stromal Cells to Apoptosis Induced by Embryonic Stimuli
Source: PLoS One. 2015 Apr 1;10(4):e0121103. doi: 10.1371/journal.pone.0121103 (PMC4382340; doi:10.1371/journal.pone.0121103)

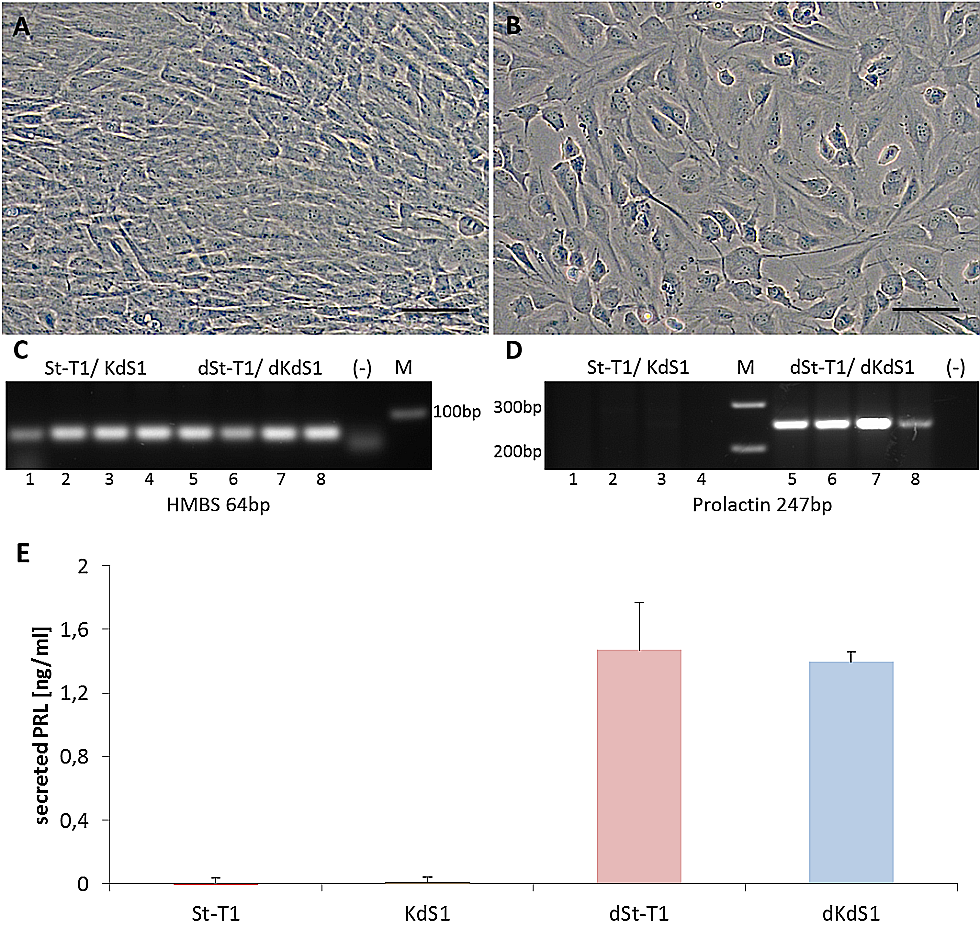

Supplement: S1 Fig — Representative bright-field microscope analysis of cell morphology before and after decidualization in vitro: (A) non-differentiated St-T1, (B) dSt-T1 after decidualization via MPA and cAMP treatment for 72h; scale bar indicates 100μm. (C) representative PCR gel for the housekeeping gene HMBS (64bp) in St-T1 (lane 1+2), KdS1 (lane 3+4), dSt-T1 (lane 5+6) and dKdS1 (lane 7+8). (D) representative PCR gel for the decidualization marker PRL (247bp) in dSt-T1 (lane 5+6) and dKdS1 (lane 7+8) vs. non differentiated St-T1 (lane 1+2) and KdS1 (lane 3+4); M = marker and (-) negative control without template. (E) quantification of secreted PRL via ELISA in in non-differentiated (St-T1, red bar; KdS1, blue bar) and decidualized (dSt-T1, bright red bar; dKdS1, bright blue bar) ESCs. Values are given as mean±SEM of n = 3 replicates. (TIF) [file pone.0121103.s001.tif]
